# Autoantibodies against NMDA receptor 1 modify rather than cause encephalitis

**DOI:** 10.1038/s41380-021-01238-3

**Published:** 2021-07-30

**Authors:** Justus B. H. Wilke, Martin Hindermann, Stefan A. Berghoff, Svenja Zihsler, Sahab Arinrad, Anja Ronnenberg, Nadine Barnkothe, Agnes A. Steixner-Kumar, Stefan Röglin, Winfried Stöcker, Michael Hollmann, Klaus-Armin Nave, Fred Lühder, Hannelore Ehrenreich

**Affiliations:** 1 Clinical Neuroscience, Max Planck Institute of Experimental Medicine, Göttingen, Germany; 2 Department of Neurogenetics, Max Planck Institute of Experimental Medicine, Göttingen, Germany; 3 Institute for Experimental Immunology, affiliated to Euroimmun, Lübeck, Germany; 4 Department of Biochemistry I—Receptor Biochemistry, Ruhr University, Bochum, Germany; 5 Institute for Neuroimmunology and Multiple Sclerosis Research, University Medical Center Göttingen, Göttingen, Germany

**Keywords:** Neuroscience, Diseases

## Abstract

The etiology and pathogenesis of “anti-N-methyl-D-aspartate-receptor (NMDAR) encephalitis” and the role of autoantibodies (AB) in this condition are still obscure. While NMDAR1-AB exert NMDAR-antagonistic properties by receptor internalization, no firm evidence exists to date that NMDAR1-AB by themselves induce brain inflammation/encephalitis. NMDAR1-AB of all immunoglobulin classes are highly frequent across mammals with multiple possible inducers and boosters. We hypothesized that “NMDAR encephalitis” results from any primary brain inflammation coinciding with the presence of NMDAR1-AB, which may shape the encephalitis phenotype. Thus, we tested whether following immunization with a “cocktail” of 4 NMDAR1 peptides, induction of a spatially and temporally defined sterile encephalitis by *diphtheria* toxin-mediated ablation of pyramidal neurons (“DTA” mice) would modify/aggravate the ensuing phenotype. In addition, we tried to replicate a recent report claiming that immunizing just against the NMDAR1-N368/G369 region induced brain inflammation. Mice after DTA induction revealed a syndrome comprising hyperactivity, hippocampal learning/memory deficits, prefrontal cortical network dysfunction, lasting blood brain-barrier impairment, brain inflammation, mainly in hippocampal and cortical regions with pyramidal neuronal death, microgliosis, astrogliosis, modest immune cell infiltration, regional atrophy, and relative increases in parvalbumin-positive interneurons. The presence of NMDAR1-AB enhanced the hyperactivity (psychosis-like) phenotype, whereas all other readouts were identical to control-immunized DTA mice. Non-DTA mice with or without NMDAR1-AB were free of any encephalitic signs. Replication of the reported NMDAR1-N368/G369-immunizing protocol in two large independent cohorts of wild-type mice completely failed. To conclude, while NMDAR1-AB can contribute to the behavioral phenotype of an underlying encephalitis, induction of an encephalitis by NMDAR1-AB themselves remains to be proven.

## Introduction

Since, the first report on a novel paraneoplastic autoimmune disease with autoantibodies against the NMDA receptor subunit NR1 (NMDAR1-AB = GluN1-AB) appeared >12 years ago [1, 2], this condition attracted considerable attention both by clinicians and basic researchers worldwide, resulting in currently nearly 2000 entries in PubMed. Variably associated with psychosis, cognitive decline, extrapyramidal symptoms, or seizures, this “NMDAR encephalitis” was already early on described to be mediated by lower surface expression of neuronal NMDAR after exposure to NMDAR1-AB of the immunoglobulin G (IgG) class [3]. The resulting NMDAR antagonistic or “ketamine-like” effects, demonstrated also in various rodent models via passive transfer of NMDAR1-AB [4–13], were interpreted as potential mechanisms of action. Thus far, however, they have failed to explain the inflammatory or encephalitic component of this condition. Interestingly, comparison of NMDAR1-AB-positive and -negative human encephalitis cases did not reveal differences, except for few perhaps NMDAR-antagonistic (ketamine-like) symptoms [14].

Even sophisticated preclinical approaches raised further questions rather than providing answers. For example, immunization of mice with liposome-embedded tetrameric NMDAR was stated to induce NMDAR-AB and fulminant encephalitis. Highly variable behavioral changes in this model included hyperactivity, immobility, circling, seizures, hunched back/lethargy, and increased mortality, including neuroinflammation, and immune cell infiltration [15], all nonspecific features of any severe encephalitis. Adequate “negative” controls like the use of other liposome-embedded holoreceptors or of control proteins of similar size would have been required to link NMDAR-AB causally with the occurrence of an encephalitis. To validate that “disease induction depends on conformationally restricted epitopes” [15], infusion of purified NMDAR-AB should have been used to clarify whether they act encephalitogenic on their own [16].

During performance of the present work, a report claimed that immunizing mice against the NMDAR1-N368/G369 region alone at high dose induced encephalitis-like behavioral and morphological impairments, including blood brain-barrier (BBB) breakdown [17]. We thus decided to follow this initially exciting report’s protocol, hoping to answer several important questions related to our own research. However, using two large independent cohorts of wild-type mice, we were unable to reproduce any of the described findings. This replication failure is now also included in the present paper.

To summarize, despite all efforts, etiology and pathogenesis of the “NMDAR encephalitis” are still as uncertain as the role of NMDAR1-AB in this condition. We note that multiple brain-directed AB apart from NMDAR1-AB have been reported in serum of healthy humans and of various other mammalian species, likely belonging to the normal pre-existing “autoimmune repertoire.” These AB do have potential functionality and also potential pathogenicity [18–22]. In fact, functional serum NMDAR1-AB of all immunoglobulin classes are a highly frequent finding across mammals, with multiple possible inducers or boosters identified thus far, ranging from genetic predisposition, various viral infections (e.g., influenza, herpes), oncological conditions, and brain lesions to chronic life stress [1, 6, 23–26]. However, no solid proof exists to date that NMDAR1-AB by themselves can induce an encephalitic process.

The present study addresses the hypothesis that “NMDAR encephalitis” may result from a primary brain inflammation coinciding with the presence of NMDAR1-AB, which ultimately shape the encephalitis phenotype. Therefore, a spatially and temporally defined" sterile encephalitis was induced in young female NexCreERT2xRosa26-eGFP-DTA ( = “DTA”) mice after immunization with a cocktail of 4 NMDAR1 peptides, also including a peptide covering the NMDAR1-N368/G369 region. Females were chosen to account for the ~4:1 female/male ratio observed in human “NMDAR encephalitis” [27]. As shown in vivo earlier, this cocktail induces functionally highly active NMDAR1-AB, leading to psychosis-like symptoms in mice with compromised BBB but no brain inflammation [28]. Here, we report that NMDAR1-AB can contribute to the behavioral abnormalities of an underlying gray-matter encephalitis, but that the multifaceted encephalitic phenotype itself, involving pyramidal neurons and their NMDAR, is nearly identical between NMDAR1-AB carriers and noncarriers.

## Materials and methods

### Mice

Mouse experiments (all C57BL/6 background) were approved by the Local Animal Care and Use Committee (LAVES, Niedersächsisches Landesamt für Verbraucherschutz und Lebensmittelsicherheit, Oldenburg, Germany) in accordance with the German animal protection law. Sample sizes were based on previous experience under consideration of the RRR-principle and technical limitations (i.e., maximum of 16 animals per IntelliCage). All experiments were performed by investigators unaware of group assignment (fully-blinded). Mice were separated by genotype and treatment to avoid inclusion effects [29] or aggressive behavior against potentially affected animals. Unless otherwise stated, mice were maintained in temperature- and humidity-controlled environments in 12 h light/dark cycle (light on at 7am) with wood-chip bedding, nesting material (Sizzle Nest, Datesand, Bredbury, UK) and, *ad libitum* food and water.

**DTA cohort:** Mice with the tamoxifen-inducible gray-matter inflammation were generated by crossing homozygous Neurod6^tm2.1(cre/ERT2)Kan^ (NexCreERT2) [30] with heterozygous Gt(ROSA)26Sor^tm1(DTA)Jpmb^ (Rosa26-eGFP-DTA) [31], resulting in double-heterozygous (DTA) mice and heterozygous NexCreERT2 littermate (control) mice lacking the DTA allele. Detailed genotyping protocols are available upon request. Experiments involving DTA mice were performed on females to account for the ~4:1 female/male ratio observed in human “NMDAR encephalitis” patients [27]. Female transgenic mice were weaned at postnatal day 21 into type IV cages (55 × 38.5 × 20.5 cm, Tecniplast, Hohenpeißenberg, Germany) in groups of 16.

**Replication cohorts** comprised male C57BL/6 J wildtype mice immunized at 8–9 weeks of age [17]. Wildtype mice were obtained from Janvier (Le Genest-Saint-Isle, France), transported to our behavior unit at 3 weeks of age, and housed in type II cages (36.5 × 20.7 × 14 cm, Tecniplast) in groups of 3–5.

### Treatments

**Immunization of the DTA cohort** was conducted as previously described [28], except that immunizations were performed on postnatal day 30. Mice were immunized with a cocktail of 4 GluN1 extracellular peptides (GluN1_35-53_, GluN1_361-376_, GluN1_385-399_, and GluN1_660-811_ coupled to keyhole limpet hemocyanin; Synaptic Systems, Göttingen, Germany) and/or chicken ovalbumin (“OVA”, A5503, Sigma-Aldrich, Darmstadt, Germany) emulsified in an equal volume of complete Freund’s adjuvant (CFA) containing 1 mg/mL heat-killed *Mycobacterium tuberculosis* H37 Ra (#231141, Difco, BD, Heidelberg, Germany) in incomplete Freund’s adjuvant. GluN1 peptide cocktail (50 µg) and/or ovalbumin (20 µg) were injected subcutaneously at the tail base.

**Immunization of the replication cohorts** followed the protocol of Wagnon et al. [17]. Male C57B/6 J mice were immunized at 8–9 weeks of age with either GluN1_168-187_ (control peptide), GluN1_359-378_ (active peptide) or for our additional comparison with ovalbumin, each emulsified in an equal volume of CFA (as described above). Antigens (200 µg) were equally distributed over 4 subcutaneous injections, 2 at shoulders and 2 at hind limbs. In addition, mice received 2 intraperitoneal injections of 200 ng of *Bordetella* pertussis toxin (#180, List Biological Laboratories) in PBS, immediately after and 48 h after immunization.

**Tamoxifen induction:** Tamoxifen (CAS#10540-29-1, T5648, Sigma) was dissolved in corn oil (C8267, Sigma) on injection days at 10 mg/mL. Mice received 2 intraperitoneal injections of 100 mg of tamoxifen/kg body weight on 2 consecutive days.

**Transponder placement:** For the experimenter-independent phenotyping of mice in the IntelliCage^®^ apparatus (TSE Systems, Bad Homburg, Germany) ISO standard transponders (8.5 mm length, 1.2 mm diameter, PM162-8) were implanted below the skin of the neck after intraperitoneal injection of 24 µL of 1.36% 2,2,2,-tribromoethanol (T48402, Sigma) in ddH_2_O/g body weight (Avertin). One week after implantation, mice were placed into IntelliCages.

**Blood sampling:** Intermediate blood samples (100 µL) were collected from the retro-orbital sinus. Terminal blood (500 µL) was sampled by cardiac puncture before transcardial perfusion. EDTA-plasma aliquots were stored at −80 °C.

### Behavioral phenotyping

**Experiments of the DTA cohort** were performed in the following order: LABORAS (baseline–prior to tamoxifen induction), bar test, hurdle test, IntelliCage-based phenotyping including pheromone preference, LABORAS, Morris water maze, hole board, prepulse inhibition (PPI), marble-burying test, and complex wheel running.

**Behavioral testing of the replication cohort** followed the design of Wagnon et al. [17]. Behavioral analyses comprised open-field activity, Y-maze working memory, elevated plus maze (anxiety), and forced-swim test (depression-like behavior). For the second replication cohort, we rearranged the test schedule to assess anxiety-like behavior in behaviorally naive mice, followed by testing in open field and Y-maze.

Except for home cage-based tests, all tests ran during the light phase. Behavioral testing was performed as previously described in detail [17, 29, 32–38]. Only home cage-based tests are briefly summarized below.

### LABORAS

To characterize the spontaneous home cage behavior of mice prior to and after a tamoxifen-induced gray-matter inflammation, the LABORAS system (Metris B.V.) was employed [32–34, 37]. Briefly, mice habituated for 2 nights to the experimental room and single housing in clear polycarbonate cages (Makrolon type I, 22 cm × 16 cm × 14 cm, Tecniplast) with wood-chip bedding, food, and water ad libitum. After two nights, cages were placed on a sensor platform (Carbon Fiber Plate 1000 mm × 700 mm × 700 mm × 30 mm, Metris B.V., Hoofddorp, Netherland) and the resulting electrical signals recorded throughout the dark phase (12 h) and classified into behavioral categories, i.e., eating, drinking, scratching, circling, climbing, immobility, locomotion, and grooming.

### IntelliCage-based phenotyping

To assess a variety of cognitive measures with minimal experimenter intervention on a 24/7 basis in a social home cage-based setting, 16 mice per group were placed in standard laboratory rodent cages (55 × 38.5 × 20.5 cm, Tecniplast) equipped with the IntelliCage^®^ apparatus (TSE-Systems) controlled by NewBehavior software (version 3.1.7), as we described in great detail previously [29]. Water was accessible in four triangular conditioning chambers (15 × 15 × 21 cm), located in cage corners. The conditioning chambers were equipped with temperature sensors and RFID antennas to identify entering mice. If mice entered a correct corner during the allocated time window, doors blocking access to water or sucrose bottles (dependent on test setting) could be opened via nosepokes sensed by light barriers. Lickometers registered licks on bottles.

The experimental setup comprised measurement of place learning (day 1), reversal learning (day 2), sucrose preference (day 3), acquisition and recollection of a place time-reward/episodic-like memory (days 4 and 5), and behavioral extinction of operant responses (days 6 and 7). General activity and day/night pattern were assessed by recording number and timing of visits to operant chambers (corner visits). On days 1–2, each mouse had access to water at a single corner (4 mice/corner), during days 3–5 to a 2% sucrose solution on one corner and water at the opposite site (8 mice/corner). On days 6–7, mice had access to only water at previously rewarded corners. In-between test sessions, corners were re allocated. The efficiency of place and reversal learning was assessed for 24 h each by calculating the percentage of place errors (visits to blocked corner/total corner visits *100%). Sucrose preference means the percentage of licks at sucrose bottle/total licks within 24 h. To evaluate episodic-like memory, access to water and sucrose solution was restricted to the first 2 h after dark-phase onset. Within this 2 h period, preference to sucrose corner was calculated as percentage of visits to sucrose corner/total corner visits.

Two weeks after this IntelliCage testing, mice returned to IntelliCages to evaluate pheromone preference using two social boxes, supplemented with fresh wood-chip bedding, connected to the left and right side of the IntelliCage via two plastic tubes, equipped with two ring RFID antennas to track individual mouse. The time each mouse spent in the IntelliCage, the neutral or the target social box was recorded along with the number of social box visits. After habituation of 1 h, social boxes were replaced by novel cages filled with either fresh wood-chip bedding (neutral site) or bedding from male C3H mice (target site) and mice again had 1 h to explore the social boxes. In addition to time spent on the target site, the time spent exploring either social box was evaluated as the readout for exploratory behavior.

### Complex wheel running

To assess locomotor activity and motor-cognitive learning, a complex running-wheel-setup (CRW) was used. Mice were single-housed in type-III cages (42 × 26 × 18 cm, Tecniplast), equipped with CRW (TSE-Systems). CRW are characterized by randomized omitted bars [39–41]. Mice were habituated to the experimental room and CRW for 2 h prior to the dark phase. After dark-phase onset, voluntary running was automatically tracked for 4 h via Phenomaster software (TSE-Systems) and the total running distance per mouse calculated.

### Antibody determinations

**ELISA:** Immunizations were confirmed by antigen ELISA, at 21–27 days after immunization in the DTA cohort and at 17–18 days in replication cohorts. ELISA was performed as described [28]. ELISA plates (96-well F-bottom Immuno MediSorp, Nunc) were coated overnight at 4 °C with either 0.2 µg ovalbumin, 0.5 µg GluN1-antigen cocktail, 1 µg GluN1_168-187_ or 1 µg GluN1_359-378_ in 50 µL PBS per well. After blocking with 5% bovine serum albumin (BSA, #8076.3, Roth) in PBS, mouse plasma (1:1000 dilution with 5% BSA/PBS 50 μl/well) was added for 2 h at RT. Antigen-specific IgG antibodies were detected using HRP-coupled anti-mouse-IgG-specific antibodies (1:10,000, A9917, Sigma) and 3,3′,5,5′-tetramethylbenzidine substrate (#555214, BD OptEIA, BD). Absorbance at 450 nm was measured and corrected for values at 620 nm by microplate reader (Tecan-Trading AG, Männedorf, Switzerland). The threshold for a positive classification was set as three standard deviations above the mean of control samples.

**Cell-based assay (CBA) for NMDAR1-AB detection:** To determine NMDAR1-AB IgG titers against full-length GluN1, a commercially available cell-based assay comprising *Grin1*-transfected and control-transfected HEK293 cells (FB 112d-1010-51, EUROIMMUN, Lübeck, Germany) was used according to the manufacturer’s instructions with minor modifications. The anti-human secondary antibody solution was replaced by Alexa Fluor 488-labeled anti-mouse IgG (1:1000, A21202, Thermo Fisher Scientific, Darmstadt, Germany) in 0.2% Tween20/PBS. Titers were determined starting at 1:100 dilutions with subsequent testing of positive samples at 1:1000 and 1:10,000 and independently evaluated by three investigators. For colocalization experiments, diluted plasma samples were spiked with a non overlapping rabbit IgG anti-GluN1 antibody directed against the C-terminal domain (1:1000, G8913, Sigma) and an Alexa Fluor 647-labeled anti-rabbit IgG antibody (1:1000, A31573, Thermo) was added to the secondary antibody solution. Images were acquired on a confocal microscope (LSM880, Zeiss, Oberkochen, Germany).

### Measurements assessing blood–brain-barrier integrity

BBB integrity was evaluated as previously described [28, 42]. Briefly, mice received intravenous injections of Evans blue (50 µg/g body weight, E2129, Sigma) and sodium fluorescein (200 µg/g body weight, F6377, Sigma). After 4 h, mice were anesthetized and transcardially perfused. Brains were collected, frozen on dry ice, and lyophilized. Tracers were extracted from hemispheres with formamide and quantified in triplicates on a fluorescent microscope (Observer Z2, Zeiss). The concentrations of tracers were calculated using a standard curve and normalized to controls.

### Histology

Mice were anesthetized with Avertin, transcardially perfused with Ringer (B. Braun, Melsungen, Germany) and subsequently 4% formaldehyde/PBS solution. Brains were collected, post fixed in 4% formaldehyde/PBS for 12 h, dehydrated in 30% sucrose/PBS for 48 h, embedded in optimal cutting medium (Tissue-Tek, #4583, Sakura, Umkirch, Germany) and frozen on dry ice. Frozen brains were cut into 30 µm coronal sections on a cryostat (CM1950, Leica, Wetzlar, Germany), and stored at −20 °C in 25% ethylene glycol/25% glycerol/PBS. Quantifications were performed on regularly spaced sections (every 300 µm) between Bregma coordinates −1.34 to −2.24 mm and 4–6 hippocampi from 2 to 3 sections were used per mouse. CA2/CA3 region is referred to as CA3 in text and figures.

Free-floating frozen sections were blocked and permeabilized for 1 h at RT with 5% normal horse serum (NHS, 26050-088, Thermo) in 0.5% Triton X-100/PBS, incubated overnight at 4 °C with primary antibodies and subsequently stained with the corresponding fluorescently labeled secondary antibodies, for 2 h at RT. Nuclei were stained for 10 min at RT with 0.2 µg/mL 4′,6-diamidino-2-phenylindole in PBS (DAPI, D9542, Sigma) and sections mounted on SuperFrost®-Plus slides (J1800AMNZ, Thermo) with Aqua-Poly/Mount (#18606, Polysciences, Warrington, PA, USA). The following primary antibodies were used: Mouse anti-GFAP (1:500, NCL-GFAP-GA5, NovoCastra-Leica, Newcastle upon Tyne, UK), guinea pig anti-S100b (1:750, #287004, Synaptic Systems), rabbit anti-Iba1 (1:1000, #019-19,741, Wako, Neuss, Germany), rat anti-CD68 (1:500, MCA1957GA, BioRad, München, Germany), rat anti-CD45 (1:100, #103101, BioLegend, Koblenz, Germany), and guinea pig anti-parvalbumin (1:1000, #195004, Synaptic Systems). The corresponding secondary antibodies included Alexa Fluor 555 anti-rabbit (1:1000, A21428, Thermo), Alexa Fluor 555 anti-mouse (1:1000, A31570, Thermo), Alexa Fluor 647 anti-mouse (1:1000, A31571, Thermo), Alexa Fluor 633 anti-guinea pig (1:1000, A21105, Thermo), Alexa Fluor 647 anti-rat (1:1000, A21247, Thermo). For Fluorojade C-staining (AG325, Sigma) of dying neurons, sections were incubated in 0.06% potassium permanganate solution for 10 min. Following a 1 min water rinse, tissue was transferred for 10 min to a 0.0001% solution of Fluorojade C, dissolved in 0.1% acetic acid. Slides were rinsed with ddH2O and dried at 60 °C. Slides were mounted with Aqua-Poly/Mount (#18606, Polysciences). Overview images of whole-brain sections were obtained on Eclipse-TI 2 epifluorescence microscope (Nikon, Düsseldorf, Germany), equipped with 4× objective (4×/0.2 NA PLAN APO #MRD00045, Nikon). For quantification, 1 µm-thick optical sections of hippocampi were acquired as tile scans on a confocal laser scanning microscope (LSM 880, Zeiss), furnished with a 40× oil objective (40 × /1.4 NA Plan-APOCHROMAT, #420762-9900, Zeiss). Image-acquisition parameters were kept constant within experiments. Quantifications and image processing were performed with FIJI-ImageJ software [43]. Iba1+ cells (mostly microglia), parvalbumin+ cells (inhibitory neurons), and CD45+ Iba1− cells (leukocytes) were manually counted. GFAP+ area was quantified densitometrically upon uniform thresholding. Cell counts and GFAP + area were normalized to quantified areas. Data from 4 to 6 hippocampi/mouse was averaged.

### Flow cytometry

Mice were anesthetized with Avertin and transcardially perfused with 40 mL of Ringer solution (B. Braun). Brains were stored on ice in 10% fetal bovine serum (FBS, #10500-064, Thermo)/DMEM (#41965, Thermo). Olfactory bulbs and brain stems were removed and brains meshed through 70 µm cell strainers. Cells were suspended in isotonic Percoll (17-0891-01, Sigma) to a final concentration of 30% and centrifuged to remove myelin. Cells were washed with FACS buffer (2% FBS, 10 mM EDTA in PBS) and filtered through 40 µm cell strainers. Fc receptors were blocked for 10 min at 4 °C with anti-mouse CD16/32 antibodies (1:100, 14-0161, Thermo). Cells were stained for 30 min at 4 °C with the following antibody mix: APC anti-CD45 (1:200, clone 104, BioLegend), PE anti-CD11b (1:200, clone M1/70, BioLegend), PECy5 anti-CD4 (1:1000, clone H129.19, BioLegend), PECy7 anti-CD8 (1:500, clone 53-6.7, Biolegend), APCCy7 anti-CD19 (1:200, clone 6D5, BioLegend), FITC anti-B220 (1:250, clone RA3-6B2, BioLegend), and PerCP-Cy5.5 anti-CD138 (1:200, clone 281-2, BioLegend). After staining, cells were washed and suspended in 400 µL of FACS buffer and 100 µL of APC quantification beads (#340487, BD). Samples were measured on a FACSAria Sorp (BD) and CytoFLEX S (Beckman Coulter, Krefeld, Germany). Cell numbers were corrected for the number of recorded APC beads. Leukocytes (CD45^high^, CD11b^low^) and microglia/macrophages (CD45^mid^, CD11b^high^) were quantified within single-cell gate determined by forward and side scatter. CD4^+^ and CD8^+^ T-cells were quantified within leukocyte gate. CD19^+^ B cells and CD138^+^ plasma cells were quantified in CD4^-^ CD8^-^ leukocyte gate.

### Statistical analysis

Statistical analyses were performed using Prism software (GraphPad Software) with the exception of the mixed ANOVA that was conducted on PPI data with R4.0.3 [44] using the rstatix package [45]. The results are presented as mean ± standard deviations (SD), with few exceptions as indicated in the figure legends. Data normality was assessed using the Shapiro–Wilk test with an alpha error of 0.05. Dependent on data distribution, two-tailed unpaired Welch’s-corrected *t*-tests or Mann–Whitney *U*-tests were used to compare groups of 2. Similarly, repeated-measure ANOVA, one-way ANOVA or Kruskal-Wallis test were used to compare multiple groups. *P* values  < 0.05 were considered statistically significant.

## Results and discussion

### *Diphtheria* toxin-mediated ablation of pyramidal neurons to mimic encephalitis affecting primarily gray matter

**Fig. 1:**
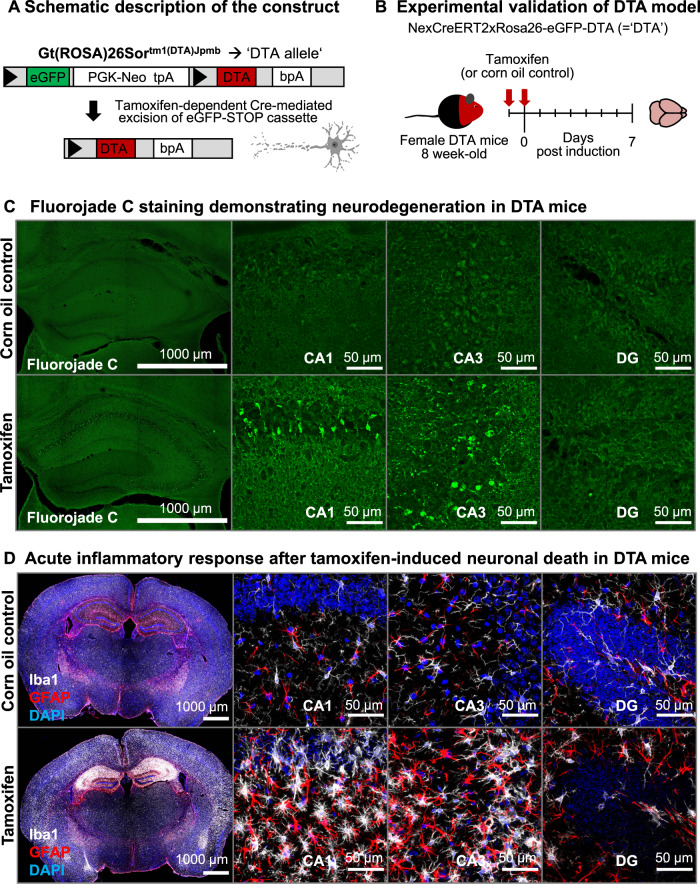
Acute inflammatory response upon tamoxifen-induced Cre recombination in NexCreERT2xRosa26-eGFP-DTA (DTA) mice. **A** Schematic description of the Rosa26-eGFP-DTA allele. Upon tamoxifen-dependent Cre translocation, a loxP- (triangles) flanked eGFP-STOP cassette is excised, resulting in expression of *diphtheria* toxin chain A and ultimately cell death. **B** Experimental validation of the acute inflammatory response in DTA mice. Female DTA mice, 8-week old, received 2 intraperitoneal injections of either tamoxifen or solvent control (corn oil). **C**, **D** Brains were collected after 7 days and stained for neurodegeneration/cell death with Fluorojade C, as well as microglia (Iba1) and astrocytes (GFAP) as indicators of reactive gliosis. High-resolution images of CA1, CA3, and dentate gyrus (DG) regions were acquired as 10 µm Z-stacks and are displayed as maximum-intensity projections.

To mimic features of a viral encephalitis in a spatially and temporally defined sterile experimental approach, we employed young female NexCreERT2xRosa26-eGFP-DTA ( = “DTA”) mice with an inducible transgene for *diphtheria* toxin-mediated ablation of pyramidal neurons, generating gray-matter inflammation [30, 31, 46]. Cell death can be controlled by dosing tamoxifen. Thus, in a series of dose-titrating pilot experiments, we selected a 2-day tamoxifen-injection design (Fig. 1A, B). After 7 days, we histologically confirmed acute neurodegeneration, pyramidal neuron loss, and a distinct inflammatory response comprising micro- and astrogliosis (Fig. 1C, D).

### Generation of primary brain inflammation coinciding with the controlled presence of NMDAR1-AB following immunization

**Fig. 2:**
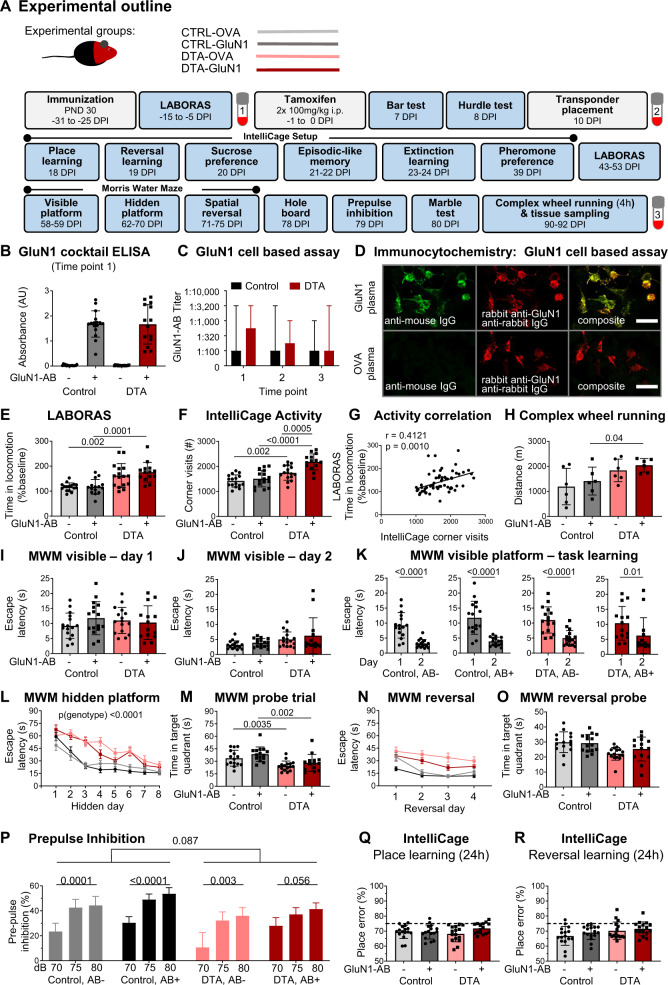
Pathophysiological relevance of NMDAR1-AB (=GluN1-AB) in the context of gray-matter inflammation. **A** Experimental outline indicating experimental groups, as well as order and time of behavioral tests (DPI = days post induction, i.e., after the last tamoxifen injection). **B** NMDAR1-AB validation by ELISA. **C** Cell-based (HEK293T) clinical standard assay for NMDAR1-AB (Euroimmun). **D** Immunocytochemical colocalization (CBA, Euroimmun) with a commercial rabbit GluN1-AB directed against the C-terminal domain. **E** Intra individual change of locomotor activity assessed in LABORAS at baseline and after tamoxifen induction. For each mouse, time in locomotion after tamoxifen induction was normalized to time in locomotion prior to tamoxifen application (baseline). **F** Activity (number of corner visits) over a 7-day IntelliCage session. **G** Pearson correlation between IntelliCage activity and intra individual changes of LABORAS locomotor activity. **H** Locomotor activity assessed by 4 h voluntary complex wheel running. **I–O** Cognitive testing in Morris water maze (MWM). **I–K** Visible platform task comprising 2 training days, demonstrating the ability for fast escape and simple task learning using within-maze cues. **L** Training of hidden platform task using extra-maze cues. DTA mice performed significantly worse than control mice (repeated-measures ANOVA, *p* < 0.0001), whereas no effect of NMDAR1-AB was observed in either DTA (*p* = 0.3273) or control mice (*p* = 0.5972). **M** Evaluation of spatial memory in the probe trial. **N** Spatial reversal of the hidden platform. **O** Evaluation of cognitive flexibility and reversal learning in a second probe trial after spatial reversal training. **P** Prepulse inhibition of acoustic startle. Intra group comparisons performed using repeated-measure one-way ANOVA; inter group comparison between genotypes for 75 and 80 dB prepulses performed using mixed ANOVA. **Q** IntelliCage-based evaluation of place learning and **R** reversal learning within 24 h sessions shows similar performance across groups. Dashed lines indicate performance at chance level (75%). Experiments were performed with 14–16 mice/group, except for CRW (*H, n* = 6 mice/group). Data presented as mean  ±  SD, except for repeated measure data (**L**, **N**, **P**; mean  ±  SEM) and AB titers (**C**, median, range).

We next addressed our hypothesis that “NMDAR encephalitis” may result from a primary brain inflammation concurring with the presence of NMDAR1-AB, which ultimately shape the encephalitis phenotype. Therefore, the above-described, defined sterile encephalitis was induced in young female DTA mice after active immunization with a cocktail of four peptides of extracellular NMDAR1/GluN1 domains, including a peptide covering the NMDAR1-N368/G369 region, versus ovalbumin as control immunization. This cocktail induces sufficient titers of highly functional NMDAR1-AB of the IgG class [28]. Active-immunization was performed at postnatal day 30, followed by an experimental scheme as detailed in Fig. 2A, comprising induction of encephalitis, extensive behavioral testing, blood sampling, and finally perfusion.

Immunization of mice against the 4 NMDAR1 peptides led to high circulating levels (even though somewhat variable between subjects) of specific AB, as shown by ELISA and cell-based assay, which persisted throughout the experiment, i.e., over 4 months. Titers did not differ between DTA mice and controls (Fig. 2B–D).

### Comparison of multidimensional behavioral readouts among DTA and control groups with or without NMDAR1-AB

Testing mice in a multifaceted battery should reveal behavioral domains affected by the induced gray-matter encephalitis and shaped by NMDAR1-AB (Fig. 2E–R; Table 1). Indeed, spontaneous home cage behavior, measured by LABORAS, and corner visits as activity readout in the IntelliCage setup, revealed considerable hyperactivity of the DTA mice, interpretable as psychosis-like behavior or “loss of inhibition” [47]. The hyperactivity measures substantially inter correlated and were more pronounced upon the presence of NMDAR1-AB (Fig. 2E–G). Additional evidence of pathological hyperactivity is presented in Table 1. In fact, this psychosis-like feature seemed to persevere, since also the final test performed before perfusion, complex wheel running, similarly indicated hyperactivity (Fig. 2H).

**Table 1:** Detailed presentation of the results obtained with the behavioral test battery in the DTA immunization model.

	Group 1 Control, OVA-AB		Group 2 Control, GluN1-AB		Comparison 1 vs. 2		Group 3 DTA, OVA-AB		Group 4 DTA, GluN1-AB		Comparison 3 vs. 4		Comparison 1 vs. 3		Comparison 2 vs. 4	
	Mean ± SD	*n*	Mean ± SD	*n*	test	*p* value	Mean ± SD	*n*	Mean ± SD	*n*	test	*p* value	test	*p* value	test	*p* value
**Health status**																
Body weight (g): pre tamoxifen	19.1 ± 0.8	16	19.5 ± 1.0	16	*t*	0.3522	18.9 ± 0.9	16	19.0 ± 0.9	14	*t*	0.6899	*t*	0.3594	*t*	0.2221
Body weight (g): 10d post tamoxifen	21.4 ± 0.8	16	21.8 ± 0.7	16	*t*	0.1694	20.8 ± 0.8	16	21.2 ± 0.9	14	*t*	0.2066	*t*	0.0372	*t*	0.0489
Body weight (g): 79d post tamoxifen	23.6 ± 0.8	16	23.5 ± 0.8	16	*t*	0.6761	23.2 ± 1.0	16	23.2 ± 0.9	14	*t*	0.9760	*t*	0.1886	*t*	0.3524
Food intake (g): pre tamoxifen	10.9 ± 1.0	16	10.5 ± 1.3	16	*t*	0.3044	10.5 ± 1.0	16	10.8 ± 1.1	14	*t*	0.3649	*t*	0.2004	*t*	0.4688
Food intake (g): post tamoxifen	11.7 ± 1.1	16	12.5 ± 1.3	16	*t*	0.0659	12.1 ± 1.7	16	12.1 ± 1.4	14	*t*	0.9875	*t*	0.4654	*t*	0.3806
Water intake (g): pre tamoxifen	13.1 ± 2.3	16	12.8 ± 1.5	16	*U*	0.8744	12.3 ± 1.8	16	13.0 ± 1.7	14	*t*	0.2818	*U*	0.4962	*t*	0.7581
Water intake (g): post tamoxifen	12.1 ± 1.3	16	12.3 ± 1.3	16	*t*	0.6492	12.3 ± 1.6	16	11.7 ± 1.2	14	*t*	0.2722	*t*	0.7220	*t*	0.1966
**Locomotor activity**																
LABORAS (baseline): Time in locomotion (s)	1798 ± 335	16	1804 ± 339	16	*t*	0.9598	1945 ± 392	16	1852 ± 712	14	*t*	0.6676	*t*	0.2613	*t*	0.8192
LABORAS (post tam.): Time in locomotion (s)	2092 ± 453	16	2096 ± 666	15	*t*	0.9836	3093 ± 872	16	3129 ± 909	14	*t*	0.9113	*t*	**0.0005**	*t*	**0.0020**
Complex wheel running: Distance (m)	1195 ± 729	6	1414 ± 556	6	*t*	0.5726	1841 ± 453	6	2045 ± 269	6	*t*	0.3691	*t*	0.1010	*t*	0.0400
IC: Total corner visits (#)	1420 ± 245	16	1506 ± 326	16	*t*	0.4071	1741 ± 288	16	2181 ± 321	14	*t*	**0.0005**	*t*	**0.0020**	*t*	**<0.0001**
IC: Corner visits in dark phase (#)	1076 ± 177	16	1136 ± 262	16	*t*	0.4547	1400 ± 270	16	1713 ± 264	14	*t*	**0.0033**	*t*	**0.0005**	*t*	**<0.0001**
IC: Corner visits in light phase (#)	344 ± 81	16	370 ± 81	16	*t*	0.3744	341 ± 85	16	468 ± 94	14	*t*	**0.0006**	*t*	0.9142	*t*	**0.0052**
Dark vs. light phase	*t*-test	***p*** < **0.0001**	*t*-test	***p*** < **0.0001**			*t*-test	***p*** < **0.0001**	*t*-test	***p*** < **0.0001**						
Number of circles (#)	774 ± 248	16	772 ± 305	15	*t*	0.9813	874 ± 393	16	849 ± 283	14	*U*	0.6896	*U*	0.6689	*t*	0.4874
**LABORAS phenotyping after tamoxifen-induction (activity)**																
Time spent climbing (s)	10274 ± 4243	16	10999 ± 6146	15	*t*	0.7075	9703 ± 3265	16	9336 ± 5312	14	*t*	0.8251	*t*	0.6726	*t*	0.4417
Time spent rearing (s)	1475 ± 373	16	1465 ± 322	15	*t*	0.9360	1668 ± 470	16	1532 ± 465	14	*t*	0.4352	*t*	0.2093	*t*	0.6570
Time spent grooming (s)	2907 ± 989	16	2768 ± 845	15	*t*	0.6780	2766 ± 820	16	2670 ± 1089	14	*t*	0.7899	*t*	0.6641	*t*	0.7887
Time immobile (s)	13124 ± 3843	16	12283 ± 6087	15	*t*	0.6522	11369 ± 3865	16	12320 ± 4191	14	*t*	0.5257	*t*	0.2075	*t*	0.9849
**Exploration**																
IC-Pheromone habituation: Time exploring (s)	2531 ± 215	16	2518 ± 203	16	*t*	0.8636	2576 ± 186	16	2591 ± 236	14	*t*	0.8531	*t*	0.5272	*t*	0.3766
Hole board: Visits (#)	28.6 ± 9.1	16	29.8 ± 13.0	16	*t*	0.7553	25.6 ± 8.3	16	21.1 ± 9.3	14	*U*	0.1075	t	0.3393	*U*	0.0680
**Marble test (stereotypy)**																
Buried marbles (#)	13.6 ± 4.4	16	13.6 ± 6.4	16	*t*	> 0.9999	12.1 ± 6.3	16	13.3 ± 7.8	14	*t*	0.6596	*t*	0.4401	*t*	0.8982
**Bar test (catalepsy)**																
Time immobile on bar (s)	0.1 ± 0.1	16	0.1 ± 0.0	14	*U*	0.3139	0.1 ± 0.1	16	0.1 ± 0.1	13	*U*	> 0.9999	*U*	0.1579	*U*	0.0426
**Hurdle test (executive function)**																
Crossings (#)	8.0 ± 2.9	16	8.8 ± 2.5	16	*U*	0.2352	8.9 ± 4.3	16	9.3 ± 3.2	14	*U*	0.4114	*U*	0.7702	*t*	0.6564
Escape latency (s)	35.3 ± 12.3	16	43.5 ± 23.0	16	*U*	0.5641	54.7 ± 33.4	16	44.3 ± 25.8	14	*U*	0.2751	*U*	0.0796	*U*	0.9510
Ratio (Latency/(Crossings+1))	4.1 ± 1.4	16	4.5 ± 2.3	16	*U*	0.7240	6.3 ± 4.5	16	4.4 ± 2.7	14	*U*	0.1661	*U*	0.1188	*U*	0.6971
**Startle**																
Startle response at 120 db (AU)	2.8 ± 2.0	16	2.3 ± 1.3	16	*U*	0.7450	2.0 ± 1.5	16	1.8 ± 0.8	14	*U*	0.5451	*U*	0.2203	*U*	0.3827
**IC Pheromone preference**																
Habituation: Time on target site (s)	1111 ± 257	16	1062 ± 141	16	*t*	0.5116	1120 ± 190	16	1236 ± 227	14	*t*	0.1430	*t*	0.9121	*t*	0.0215
Test phase: Time on target site (s)	1548 ± 382	16	1584 ± 288	16	*t*	0.7674	1675 ± 211	16	1682 ± 375	14	*t*	0.9489	*t*	0.2581	*t*	0.4337
Habituation vs. Test	*t*-test	***p*** < **0.0008**	*t*-test	***p*** < **0.0001**			*t*-test	***p*** < **0.0001**	*t*-test	***p*** < **0.00010**						
**IC Sucrose preference (anhedonia)**																
Preference to sucrose bottle (%)	99.4 ± 0.7	16	98.9 ± 1.1	16	*U*	0.3737	99.8 ± 0.3	16	99.6 ± 0.6	14	*U*	0.5929	*U*	**0.0112**	*U*	0.0519
**IC phenotyping (place & reversal learning)**																
Place learning: Place error (%)	69.5 ± 4.4	16	69.4 ± 4.6	16	*t*	0.9623	68.2 ± 5.3	16	71.8 ± 3.7	14	*t*	0.0345	*t*	0.4465	*t*	0.1241
Reversal learning: Place error (%)	66.8 ± 6.2	16	69.0 ± 4.6	16	*t*	0.2599	70.2 ± 5.8	16	71.7 ± 4.5	14	*U*	0.2572	*t*	0.1167	*t*	0.1204
**IC phenotyping (What-Where-When memory):** Preference to (former) sucrose corner (%)																
Acquisition	27.0 ± 7.8	16	30.5 ± 6.1	16	*t*	0.1605	24.1 ± 10.2	16	30.1 ± 7.3	14	*t*	0.0728	*t*	0.3790	*t*	0.8637
Retrieval	37.8 ± 9.2	16	40.2 ± 9.8	16	*t*	0.4805	34.5 ± 13.9	16	43.0 ± 8.4	14	*t*	0.0505	*t*	0.4345	*t*	0.4092
day1 vs day2	*t*-test	***p*** < **0.0013**	*t*-test	***p*** < **0.0026**			*t*-test	***p*** < **0.0234**	*t*-test	***p*** < **0.0002**						

*P* values withstanding Bonferroni correction (α < 0.0125) are bolded.

*IC* intelliCage, *t* two-sided Welch’s corrected *t*-test, *U* two-sided Mann–Whitney *U*-test.

For hippocampal learning and memory, the classical Morris water maze (MWM) test was employed [48]. Whereas the visible platform days showed comparable ability of task learning among groups (Fig. 2I–K), substantial deficits arose regarding the hidden platform learning curves (Fig. 2L) and the probe trial results (Fig. 2M). Here, DTA mice, independent of the presence of NMDAR1-AB, demonstrated inferior performance consistent with hippocampal damage. This was also seen as a clear tendency in MWM reversal testing as hippocampus-dependent cognitive flexibility measure, again without an appreciable NMDAR1-AB effect (Fig. 2N–O).

Prepulse inhibition of the startle response (PPI) was employed as a surrogate marker for gating defects in the prefrontal cortex–known also as a highly relevant translational test in human patients with psychosis. PPI deficit is among the most reliable objective features of severe neuropsychiatric phenotypes, likely affecting the ability to adequately filter and interpret environmental stimuli [47, 49–52]. Indeed, DTA mice, again independent of NMDAR1-AB, showed an overall tendency of a PPI deficit, pointing to prefrontal cortical network dysfunction as a consequence of the induced pyramidal neuronal death (Fig. 2P).

To some surprise, our previously designed extensive cognitive, emotional, and social phenotyping of mice in an observer-independent setting, using IntelliCages [29], did not reveal any considerable differences, except for the above-delineated distinct hyperactivity. This is most likely explained by lower sensitivity or ceiling effects of this paradigm (requiring a higher level of damage to show impairment), and the just partial involvement of hippocampal functions that are apparently fully compensated (example of IntelliCage readouts in Fig. 2Q, R). Similarly, all other behavioral tests failed to show appreciable alterations. Few just marginally significant and sometimes rather counter intuitive results proved invalid upon multiple-testing correction (Table 1). In conclusion, behavioral testing uncovered the expected consequences of pyramidal neuronal degeneration, i.e., substantial hippocampal dysfunction (MWM), a strong tendency of gating deficits as prefrontal cortex measure (PPI), and lasting psychosis-like hyperactivity, with only the latter augmented by the presence of NMDAR1-AB.

### BBB integrity and measures of chronic inflammation in DTA and control groups with or without NMDAR1-AB

**Fig. 3:**
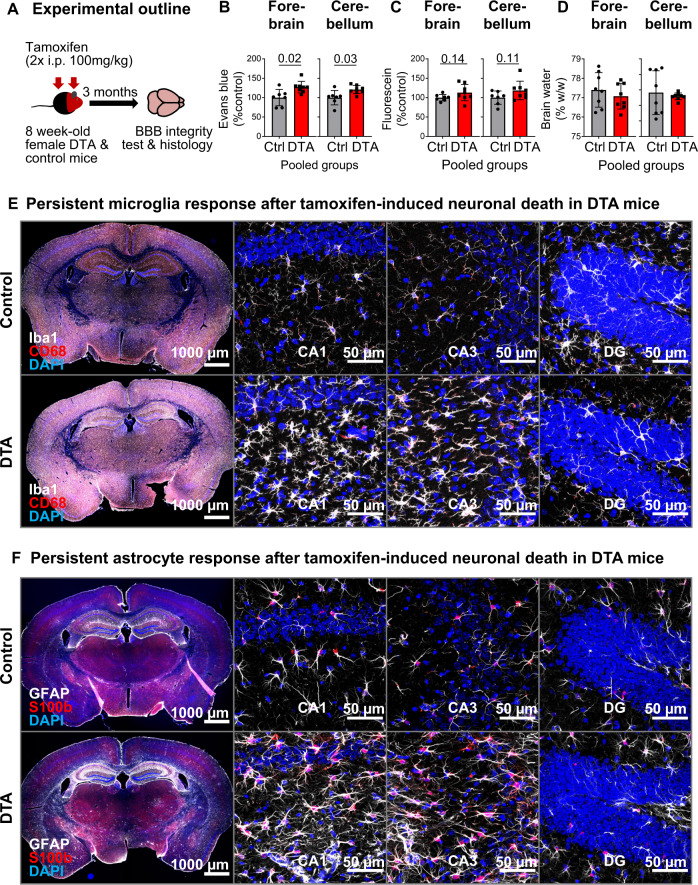
Long-lasting inflammatory response at 3 months after tamoxifen induction. **A** Abbreviated experimental outline (detailed schematic in Fig. 2A). Blood–brain barrier (BBB) permeability, irrespective of NMDAR1-AB status, assessed by Evans blue (**B**) and fluorescein (**C**) extravasation, as well as brain-water content (**D**) in the forebrain and cerebellum of DTA and control mice. Data presented as mean  ± SD. **E**, **F** Representative images demonstrating persistent inflammatory changes in the hippocampus of DTA mice, including increased microglia density and apparent changes in morphology (**E**, quantifications in Fig. 4B) and increased expression of glial fibrillary acidic protein, GFAP (**F**), quantified in Fig. 4C). High-resolution images of CA1, CA3, and dentate gyrus (DG) regions were acquired as 10 µm Z-stacks and displayed as maximum-intensity projections.

Using fluorescent tracers, as described in detail previously [28, 42], we addressed BBB leakiness in our DTA encephalitis model as a prerequisite for substantial NMDAR1-AB transfer to the brain and exertion of measurable effects. Tracer extravasation was enhanced for Evans blue and displayed a strong trend for fluorescein, confirming persistent BBB dysfunction. Brain water-content data showed remarkable scatters but were comparable in DTA and control mice at 3 months after encephalitis induction (Fig. 3A–D).

**Fig. 4:**
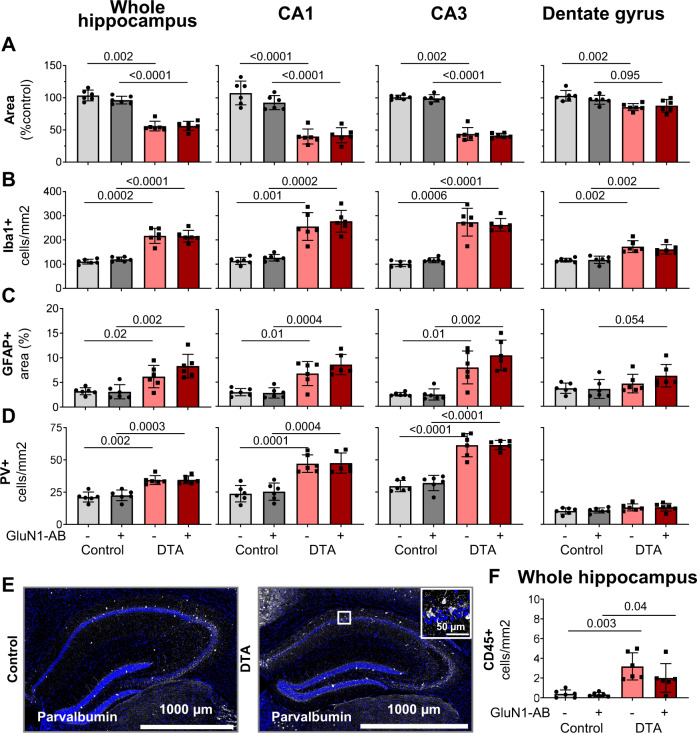
Histological quantification of typical encephalitis readouts in the hippocampus. **A** For assessment of atrophy, the respective hippocampal areas were normalized to the mean of control mice. Normalized area shows the average of 6 sections per mouse (within Bregma −1.34 mm and −1.64 mm). **B** Number of Iba1+ cells (microglia). **C** GFAP+ area determined densitometrically upon uniform thresholding. **D** Density of parvalbumin (PV)-positive interneurons. **E** Representative images of parvalbumin stainings in control and DTA mice. **F** Leukocyte (CD45+ and Iba1− cells/mm^2^) infiltration into the hippocampal parenchyma. Data from 6 mice/group displayed as mean  ± SD.

The chronic microglia and astrocyte response after tamoxifen-induced neuronal death in DTA mice was slightly milder as compared with the acute situation (see Fig. 1C, D), but still very obvious already from overview images (Fig. 3E, F). Histological quantification yielded distinct reductions of the areas of whole hippocampus, CA1 and CA3, whereas the dentate gyrus just revealed a similar tendency (Fig. 4A). Both Iba1 and GFAP quantifications followed the same pattern: strong microgliosis and astrogliosis in the whole hippocampus, CA1 and CA3, but only moderate in dentate gyrus of both DTA groups, with no significant differences in NMDAR1-AB carriers (Fig. 4B, C). NMDAR1-immunized control mice showed no signs of inflammation as reported previously [28]. Change of inhibition could be demonstrated by the relative increases in parvalbumin-positive interneurons upon DTA-induced encephalitis, sparing the dentate gyrus. Also regarding these interneurons, NMDAR1-AB presence did not modulate the ensuing picture (Fig. 4D, E). Hippocampal immune cell infiltration, determined by counting CD45 + cells, was modest but clear in DTA mice (Fig. 4F). Collectively, these data document persistent BBB disruption and distinct chronic inflammation in our DTA model but no obvious amplifying influence of NMDAR1-AB on any of these readouts. This additionally argues against any appreciable proinflammatory role of NMDAR1-AB of the IgG class.

### Failure to induce any signal of encephalitis by immunizing mice solely against the NMDAR1-N368/G369 region

**Fig. 5:**
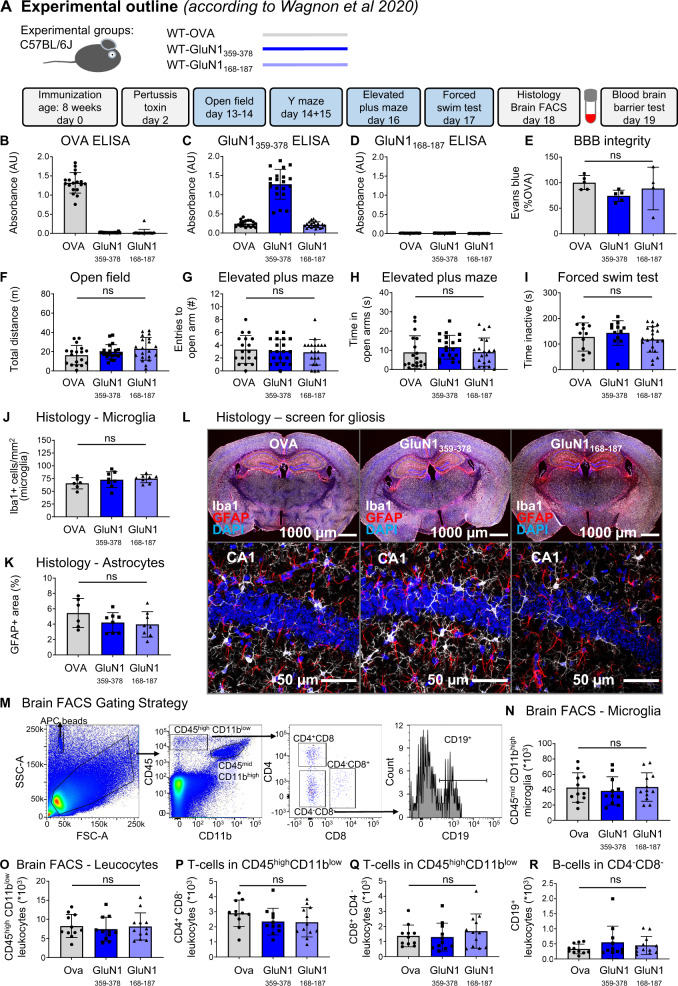
Summary of the results from the replication study. **A** Experimental outline, following the protocol of Wagnon et al. [17]. **B–D** Experimental validation of immunization success using OVA-ELISA, GluN1_359–378_-ELISA, and GluN1_168-187_-ELISA. **E** Blood–brain-barrier (BBB) integrity assessed through Evans blue extravasation. **F–I** Results of behavioral phenotyping, showing locomotor activity in the open field, anxiety-related behavior in elevated plus maze, and depression-like behavior in the forced-swim test. **J–L** Histological quantification using 8 mice/immunization with focus on reactive gliosis, showing microglia numbers, GFAP+ area (densitometry), and representative images of quantified stainings. High-resolution images of CA1 were acquired as 10 µm Z-stacks and displayed as maximum-intensity projections. **M–R** Characterization of the brains’ immune cell compartment by flow cytometry of 11–12 mice/group. **M** Gating strategy. Quantification of CD11b^high^CD45^mid^ cells (microglia). Quantification of CD11b^low^CD45^high^ leukocytes, CD4^+^ T cells, CD8^+^ T cells, and CD19^+^ B cells. Data displayed as mean ±SD.

During performance of the present work, a report claimed that immunizing mice against the NMDAR1-N368/G369 region alone induced an encephalopathy with remarkable B-cell response, provoking an autoimmune reaction against NMDAR, and encephalitis-like behavioral deficiencies [17]. We were first excited and wondered whether this model would finally help us to answer several burning questions of our own research, addressing for instance, the cellular–molecular mechanisms of NMDAR1-AB-mediated brain inflammation, which we had not been able to observe [28], or late consequences of an autoimmune encephalitis, or reasons for NMDAR1-AB titer fluctuations including the occurence and nature of late boosters [53]. Therefore, we started an extensive experiment, ultimately using two large independent cohorts of wild-type mice, and followed meticulously this report’s protocol [17]. However, as detailed in Fig. 5 and Table 2, none of the described findings could be reproduced, although the adopted immunization protocol worked well. We found specific GluN1_359-378_-AB of the IgG class in serum and no immune response using the non immunogenic control peptide GluN1_168-187_ (Fig. 5A–D). There was no BBB disturbance (Fig. 5E), all behavioral tests were normal (Fig. 5F–I), histology did not show any abnormalities (Fig. 5J–L), and brain FACS results were physiological and did not reveal any differences between groups (Fig. 5M–R). Therefore, a second mouse cohort was employed to exclude potential by-chance failure of replication but, disappointingly, yielded the same overall negative results (data not shown).

**Table 2:** Detailed presentation of behavior results obtained in replication study (protocol following Wagnon et al. [17]).

	**OVA**		**GluN1**_**359-378**_		**GluN1**_**168-187**_		**ANOVA**	
	**Mean** **±** **SD**	***n***	**Mean** **±** **SD**	***n***	**Mean** **±** **SD**	***n***	**test**	***p*** **value**
**Health status**								
Pre immune: Body weight (g)	24.2 ± 1.0	18	24.5 ± 0.9	20	24.6 ± 1.1	20	1way	0.4515
Post immune (+7d): Body weight (g)	23.3 ± 1.2	18	23.0 ± 0.8	20	23.4 ± 1.0	20	1way	0.3503
**Depression-like behavior**								
FST total time immobile (s)	127.8 ± 54.9	12	143.4 ± 48.0	12	118.2 ± 49.8	20	1way	0.4048
**Locomotor activity**								
Open field: Total distance (m)	16.3 ± 10.0	18	19.8 ± 7.5	20	23.0 ± 11.8	20	1way	0.1284
**Activity**								
Elevated plus maze: Total arm visits (#)	11.4 ± 4.0	18	11.7 ± 4.7	20	10.5 ± 5.1	20	KW	0.8547
Y maze (habituation): Total arm visits (#)	10.0 ± 4.0	18	13.3 ± 5.8	19	13.9 ± 6.9	19	1way	0.0956
**Anxiety-related behavior**								
Open field: Escape latency (s)	56.1 ± 47.1	18	27.8 ± 16.0	20	40.3 ± 40.5	20	KW	0.1941
Open field: Time in periphery (s)	376.9 ± 31.9	18	383.2 ± 22.2	20	378.3 ± 23.8	20	KW	0.7324
Open field: Time in intermediate zone (s)	33.0 ± 26.7	18	27.7 ± 17.3	20	32.6 ± 18.7	20	KW	0.6139
Open field: Time in center (s)	9.5 ± 8.0	18	8.4 ± 9.1	20	8.3 ± 6.7	20	KW	0.6410
Elevated plus maze: Visits to open arm (#)	3.3 ± 2.2	18	3.1 ± 1.8	20	2.9 ± 2.0	20	1way	0.7994
Elevated plus maze: Time in open arm (s)	9.0 ± 8.5	18	11.6 ± 6.1	20	9.1 ± 7.3	20	KW	0.2046
**Y maze (habituation, 5** **min)**								
Y maze: Visits arm 1 (#)	4.4 ± 2.4	18	6.4 ± 3.5	19	7.4 ± 4.5	19	KW	0.1004
Y maze: Visits arm 2 (#)	5.6 ± 2.5	18	6.9 ± 3.0	19	6.5 ± 2.9	19	1way	0.3567
arm1 vs. arm2	*U* test	*p* = 0.1120	*t* test	*p* = 0.5860	*t* test	*p* = 0.4426		
**Y maze (test phase, 3** **min)**								
Y maze: Visits arm 1 (#)	1.9 ± 1.6	18	2.5 ± 1.8	19	2.1 ± 2.1	19	KW	0.5813
Y maze: Visits arm 2 (#)	2.4 ± 2.6	18	2.6 ± 1.7	19	2.1 ± 1.2	19	KW	0.4752
Y maze: Visits novel arm (#)	2.8 ± 1.7	18	3.5 ± 2.2	19	3.3 ± 1.8	19	KW	0.5482
1way ANOVA	*p* = 0.2367		*p* = 0.3093		***p*** = **0.0454**			
novel vs. arm1	*U* test	*p* = 0.1403	*U* test	*p* = 0.1728	*U* test	***p*** = **0.0364**		
novel vs. arm2	*U* test	*p* = 0.1479	*U* test	*p* = 0.2151	*U* test	***p*** = **0.0334**		

Significant *p* values (α < 0.05) in bold.

*FST* forced swim test, *1way* one-way ANOVA; *KW* Kruskal-Wallis test, *t* two-sided Welch’s corrected *t*-test, *U* two-sided Mann–Whitney *U*-test.

Simultaneous with our anticlimactic replication failure, Ding et al. published a study, using a similar immunization strategy to investigate the pathogenicity of various GluN1 peptides (each also at a dose of 200 µg), including the GluN1_359–377_. Yet, in contrast to the claims of Wagnon et al., the authors did not find GluN1_359-377_-AB in the CSF of immunized mice [54]. In addition, these authors validated the functionality of GluN1_356-385_-specific AB and assessed the in vivo consequences after triple immunization in combination with pertussis toxin, resulting in a behavioral phenotype (impaired social memory and novel object recognition, normal anxiety-, and depressive-like behavior), distinct from the one reported by Wagnon et al. (impaired spatial memory, abnormal anxiety-, and depressive-like behavior) [17, 54]. Unfortunately, Ding et al. did not investigate histopathological consequences of their immunization strategy, hence, it remains unclear whether they induced any features of an encephalitis. Another active immunization model, lately published as preprint, focused on the chronic effects of NMDAR1-AB upon immunization against GluN1_402–421_ peptide. Similar to our experience with these immunizations, mice remained healthy and did not develop any encephalitic signs, despite the presence of persistent and functional NMDAR1-AB. The only behavioral alteration reliably observed in GluN1_402–421_-immunized mice was impaired spatial memory and/or novelty recognition, assessed as spontaneous alternation in T-maze [55]. These findings are highly interesting since they underscore the possibility that chronic presence of circulating NMDAR1-AB can mildly modulate behavior, even in the absence of an appreciable BBB dysfunction. This is in line with a previous expert review [18] and our experimental observation that NMDAR1-AB can reach the brain to bind there at low titers even in healthy wild-type mice [26].

We are aware that—for comparison—in experimental autoimmune encephalitis (EAE), fluctuations can occur regarding the severity of the clinical/pathological picture [56–58]; however, the absence of all claimed readouts as obtained in our replication approaches, is unheard of and quite surprising. This is more so, since the message of the report by Wagnon et al. will remain, if not questioned, and be taken for granted. This in turn can become an ethical issue, leading to (pre)clinical conclusions that are ultimately damaging for other scientists and unfortunately also for patients, as we observe on a regular basis.

### Conclusions from the NMDAR1-immunized DTA model: NMDAR1-AB modify rather than cause an encephalitis

Using a well-standardizable, spatially and temporally defined mouse model of viral encephalitis by employing controlled DTA induction in pyramidal neurons, we find a multifaceted encephalitic phenotype, which persists over months. This phenotype involves pyramidal neurons and thus of course also their NMDAR, but is only marginally aggravated in NMDAR1-AB carriers versus non carriers. The aggravation essentially rests on hyperactivity as psychosis-like behavioral readout but is not reflected in any histological quantification. Similarly, comparison of NMDAR1-AB-positive and -negative human encephalitis cases did not reveal appreciable differences, except for few NMDAR-antagonistic (ketamine-like) symptoms [14]. Most likely, human “NMDAR encephalitis” is simply not a separate condition, but rather marks an encephalitis where the highly prevalent NMDAR1-AB and/or the respective B cells happen to be present in the brain and shape the clinical picture. Therefore, it may be problematic, if the search for encephalitis causes stops after detection of NMDAR1-AB (of the IgG class). Reassuringly, “polypragmatic” treatment of any encephalitis of unknown origin (constituting the majority of cases) should anyhow include antibiotics, antivirals, and eventually corticosteroids/immunosuppressants on top of supporting measures.

The N-terminal domain containing the G7 epitope (N368/G369) was first deemed pathognomonic for “NMDAR encephalitis,” and believed to be the target region of the pathological NMDAR1-AB of the IgG class seen responsible for this condition [59]. Therefore, the respective immunization model, leading to encephalitis as described by Wagnon and colleagues [17], seemed attractive at first view and worthwhile pursuing. Unfortunately, it was not reproducible in our hands, and is not supported by a similar recent paper [54]. Searching for an explanation by speculating about possible reasons why Wagnon and colleagues found signs of an encephalitis that we did not see, interfering “iatrogenic” issues may be worthwhile considering, like e.g., undetected subclinical infections in their animal facility, leading to “occult” brain inflammation. Also, other factors, e.g., differences in the gut microbiota, which have the potential to modulate disease progression in EAE models [60], cannot be entirely excluded. However, it seems rather unlikely that the overall discrepancy, including BBB dysfunction, can be explained by such physiological factors.

Considering our own previous findings on epitopes recognized by the highly frequent NMDAR1-AB found in human serum, the negative outcome of the replication attempts is actually not too surprising. Epitope mapping using 7 different NMDAR1 constructs revealed recognition by NMDAR1-AB-positive sera of different epitopes, located in the extracellular ligand-binding and the N-terminal domain, as well as the intracellular C-terminal and the extended pore domain. NMDAR1-AB seropositivity was polyclonal/polyspecific in half of the investigated sera and likely mono- or oligoclonal/oligospecific (mainly IgG) in the other half. Overall, no particular disease-related pattern appeared. NMDAR1 epitopes were comparable across health and disease [61]. Also, the accentuated role of IgG in “NMDAR encephalitis” is still a matter of speculation, but likely related to inflammation-induced class switch in the brain [62]. As mentioned in the Introduction, NMDAR1-AB are only one of many possible autoantibodies directed against brain epitopes [18–22]. The finally resulting phenotype would then depend on (i) the specific site(s) of brain inflammation, with either resident plasma cells producing AB or a local extent of BBB breach at that site (to allow sufficient AB transfer to the brain), and (ii) the specific circulating brain-reactive autoantibody profile of each individual.

To conclude, while NMDAR1-AB can contribute to the behavioral phenotype of an underlying encephalitis, there is no proof at present for induction of an encephalitis by NMDAR1-AB themselves. Thus, based on the results presented here, the answer to the question of whether or not NMDAR1-AB can, by themselves, induce encephalitis is probably no, with the caveat that perhaps it may be possible in extremely rare patients with an exceptionally high NMDAR1-AB titer. However, this would assume that a very high titer is somehow linked to brain inflammation (as cause or consequence) and/or local increases in BBB permeability, which is neither supported by the data from this nor from other studies.

## Data Availability

All data are available upon request.
